# Edge and Cloud Collaborative Entity Recommendation Method towards the IoT Search

**DOI:** 10.3390/s20071918

**Published:** 2020-03-30

**Authors:** Ruyan Wang, Yuzhe Liu, Puning Zhang, Xuefang Li, Xuyuan Kang

**Affiliations:** 1 School of Communication and Information Engineering, Chongqing University of Posts and Telecommunications, Chongqing 400065, China; wangry@cqupt.edu.cn (R.W.); liuyuzhe95632@163.com (Y.L.); lixuefang124@163.com (X.L.); kangxuyuan163@163.com (X.K.); 2 Key Laboratory of Optical Communication and Networks, Chongqing 400065, China; 3 Key Laboratory of Ubiquitous Sensing and Networking, Chongqing 400065, China

**Keywords:** IoT search, edge computing, edge-cloud collaboration, recommendation algorithm, entity identification

## Abstract

There are massive entities with strong denaturation of state in the physical world, and users have urgent needs for real-time and intelligent acquisition of entity information, thus recommendation technologies that can actively provide instant and precise entity state information come into being. Existing IoT data recommendation methods ignore the characteristics of IoT data and user search behavior; thus the recommendation performances are relatively limited. Considering the time-varying characteristics of the IoT entity state and the characteristics of user search behavior, an edge-cloud collaborative entity recommendation method is proposed via combining the advantages of edge computing and cloud computing. First, an entity recommendation system architecture based on the collaboration between edge and cloud is designed. Then, an entity identification method suitable for edge is presented, which takes into account the feature information of entities and carries out effective entity identification based on the deep clustering model, so as to improve the real-time and accuracy of entity state information search. Furthermore, an interest group division method applied in cloud is devised, which fully considers user’s potential search needs and divides user interest groups based on clustering model for enhancing the quality of recommendation system. Simulation results demonstrate that the proposed recommendation method can effectively improve the real-time and accuracy performance of entity recommendation in comparison with traditional methods.

## 1. Introduction

With the large-scale deployments of intelligent sensing devices, it is increasingly difficult to accurately obtain interest information [1] in such a huge and complicated Internet of things. Therefore, internet of things (IoT) search technology emerges. IoT search [2] refers to obtaining various structured entity information (such as objects, people, web pages, etc.) from the physical world by adopting appropriate methods to store and order the obtained entity information [3], which is convenient for users to search. However, due to the massiveness and heterogeneity of physical entity information [4] in the Internet of things [5] and high real-time search [6] requirements, the traditional Internet search models [7,8] are inapplicable to the IoT search [9]. Search engines bridge users and applications with required resources [10]. The sensors collect entity information, and users can search the entity information in real time through a search engine. For example, search real-time traffic information to make reasonable travel plans. Alternatively, search for the number of people waiting in line at a coffee shop to choose the right time to buy coffee.

Search techniques, as the fundamental to IoT, are faced with numerous challenges [11] like a large number of devices, dynamic availability, restrictions on resource use, real-time data in various types and formats. Hence, it is desirable for users that IoT search engine is intelligent and can provide more satisfying search experience, comprehensive and personalized search results. Presently, various Internet applications have adopted recommendation algorithms to push contents or products that users may be interested [12] in according to their preferences, for example, Sina Weibo and Taobao. Due to the complexity of the physical entity information, filtering out the uninterested information from mass information only by users is extremely insufficient. It not only influences the search experience but also lowers the search accuracy. In addition, the state of physical entity is time-varying. Thus, if the response unit of the search system is far away from the user then the user obtains the required entity state information, the state information of the entity may have changed already.

Therefore, it is necessary to study the recommendation algorithm in the IoT search system. By recommending the entity status information to the user in real time, it can skip the search process, helping the user to acquire the status of the entity, recommending related entities [13] based on user interests [14], and providing references to users about how they can choose entities, which is significant to improve user experience and ensure real-time search. However, due to the time-varying characteristics of physical entities, the traditional Internet information recommendation method is unsuitable for the IoT search system.

The state data of physical entities, the characteristics of user search, and the combination of the benefits of cloud and edge to design physical entity recommendation methods have not been considered in current research. The time-varying state of physical entities is different, and users have various interests in accessing diverse entities in different time periods. Storing all entity state data on the edge or in the cloud fails to meet the accuracy and delay performance of IoT search. Therefore, an edge-cloud collaborative entity recommendation method is proposed. The main contributions of this paper are listed as follows:

(1) According to the real-time and accuracy requirements of IoT search, considering the characteristics of the differentiated distribution of physical entity state, an edge and cloud collaborative entity recommendation method (ECCRM) for IoT search is proposed. Combining with the advantages of cloud computing and edge computing, the edge and cloud collaborative entity recommendation architecture suitable for the IoT search is designed to improve the efficiency of recommendation.
(2) An edge-oriented entity recognition method is presented to accurately distinguish entities into two categories, hot and cold entities, and hot entity state information with strong time variability and high accessibility are stored in the edge server, and cold entity state information with weak time variability and low accessibility is cached in the cloud to ensure the real-time and accuracy performance of recommendation system so as to improve the user search experience.
(3) Aiming at the potential entity information acquisition needs of users, considering the user’s respective preferences, an interest group-based collaborative filtering (IGCF) recommendation algorithm is devised. A clustering algorithm is adopted to divide multiple user interest groups. By calculating the user similarity, and then recommendation results that meet the intelligent needs of users can be obtained.

The rest of this paper is organized as follows: Section 2 introduces the related work. Section 3 describes the system architecture. Section 4 proposes an active recommendation system. Section 5 proposes an active recommendation system. Section 6 verifies the proposed methods. Section 7 concludes this paper.

## 2. Related Work

In recent years, in order to ensure the timeliness of IoT search results, researchers have proposed some achievements to solve the problem of IoT search based on cloud computing. Hou et al. [15] implemented data transmission function of the Internet of Things under the Internet of Things-Cloud architecture by combining HTTP and MQTT. Zhou et al. [16] established a database in the cloud to store data collected by sensors to facilitate user search. Michel et al. [17] designed a cloud computing-based IoT search and discovery method, which optimized the scheme for querying the state information of neighboring entities. Ali et al. [18] proposed a secure data sharing in clouds (SeDaSC) solution, which encrypted data and stored it into the cloud. Users can search data from the cloud after submitting access requests to the server. Zhang et al. [19] proposed a hierarchical search solution (LHPM). After receiving the user’s search request, the gateway returned the search results to the user by finding the corresponding appropriate sensors. However, the limitation on the computing, communication and storage capacity of the sensor, and the long communication distance between the sensor and the cloud [20], lead to a large search delay.

The idea of edge computing has inspired new solutions to the information acquisitions of state time-varying entities. ECC believes that cloud computing is suitable for non-real-time, long-periodic data. However, edge computing has great advantages in real time, short-period data, and local decision-making scenarios. Mollah et al. [21] pointed out that the edge server could be used as an intermediary for the communication between the sensor and the cloud, and then interact with the cloud after processing localized data at the edge, thus improving the efficiency of search. Li et al. [22] built a deep learning model at the edge to calculate and process the data obtained from the sensors. Hossain et al. [23] used edge computing to minimize the processing delay of IoT heterogeneous data. Tang et al. [24] proposed a collaborative edge-cloud cache framework based on the STK-tree(SKIN+STK),which received search requests and cached data via an edge server. Xu et al. [25] proposed a multi-object tracking algorithm, tracked target with the help of edge computing. Fu et al. [26] proposed a secure data storage and searching framework by integrating fog computing and cloud computing, which was suitable for the Industrial IoT.

Since the idea of IoT search has been proposed, the research on recommendation methods of IoT search is relatively scarce. Forestiero et al. [27] built a distributed recommendation system by considering relationships between things, representing things by bit vectors, and managing thing identifiers by network proxies to achieve an efficient entity recommendation system. Yao et al. [28] proposed a framework based on probability factors. By integrating users and users, users and things, and the relationship between things, a hypergraph was obtained to construct the model and complete the recommendation. Yao et al. [29] summarized and elaborated the research of recommendation systems in IoT during recent years, and highlighted the unique challenges brought by IoT, at last, provided a reference solution to the next-generation smart IoT-based systems.

In conclusion, the recommendation system of physical entities is not only related to the characteristics of physical entities, but also related to the interests and preferences of users. Therefore, it is not only necessary to minimize the delay of data acquisition based on the time-varying characteristics of physical entities, but also necessary to explore the potential preferences and needs of users and recommend the required entity state information for users in real time. Hence, we designed an entity recommendation system architecture based on the collaboration between edge and cloud. Then, we presented an entity identification method suitable for edge, which takes into account the feature information of entities and carries out effective entity identification based on the deep clustering model, so as to improve the real-time and accuracy of entity state information search. Furthermore, we devised an interest group division method applied in cloud, which fully consider user’s potential search needs and divides user interest groups based on clustering model for enhancing the quality of recommendation system.

## 3. System Architecture

**Figure 1:**
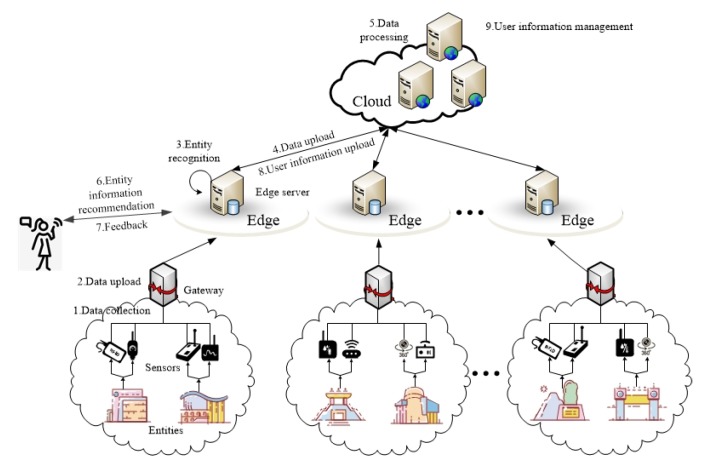
Recommendation system architecture.

The architecture proposed in this paper is as shown in Figure 1. The IoT recommendation system is divided into three layers. The first layer is the smart entity layer. The sensors attached to the entity collect and upload the state information of the entity. The second layer is the edge layer, which is composed of edge servers. The edge server uses the entity identification method mentioned below to classify entities based on the collected entity state information. The third layer is the cloud layer. The cloud server integrates user information and manages all of edge servers.

In the traditional IoT search mode, the user directly communicates with the cloud center server to issue a search request to obtain the search results. After receiving the request, the cloud center server needs to traverse the candidate sensors to obtain the state information of the entity. Because the communication distance between the user and the cloud center server is relatively long, when searching for entities with high time variability, the results received by the user from the cloud center server can no longer accurately reflect the current state of the entity. However, the edge server is close to users and entities. Compared with traditional search methods, obtaining entity state information via edge servers can greatly ensure the timeliness of entity state information obtained by users.

The proposed edge and cloud collaborative recommendation method is as follows.

(1) After the sensors that associated with the smart entity collect data, the collected entity state information is periodically uploaded to the gateway, and each gateway is responsible to manage multiple smart entities within its coverage.
(2) Each gateway uploads the status information of the smart entity to its upper-level edge server.
(3) After receiving the entity state information uploaded by the gateway, the edge server uses the proposed entity recognition algorithm to identify and classify the entity in combination with the historical entity state data and local search records.
(4) The edge server stores the hot entities with high time variability and high accessibility identified in the edge server, and uploads the state information of cold entity with low time variability and low accessibility to the cloud.
(5) The cloud server stores and processes the state information of cold entities uploaded by the edge server.
(6) The edge server responds to the user. If the user is interested in the hot entity state information that was stored in the edge server in advance, it recommends the entity state information to the user, otherwise it downloads the cold entity state information that the user is interested in from the cloud and pushes it to the user.
(7) The user sends feedback information to the edge server based on the received recommendation results.
(8) The edge server uploads user’s log information to the cloud server.
(9) Integrate user information in the cloud and use the proposed collaborative filtering algorithm based on interest groups to obtain entity recommending lists to complete the recommendation process.

In our architecture, data security is an issue that should not be ignored. For both parties to data interaction, the security of their identities needs to be verified. Due to the limited resources of sensors, a lightweight authentication scheme is needed, moreover, a trade-off between efficiency and security is required [30]. For users, a strong security user authentication scheme is needed to protect the security and privacy of communications. In our scheme, the computing ability of the sensors is limited, while users, gateways, and servers all have strong computing ability. Therefore, when uploading data, the sensor authenticates with its unique device information. Each time when search, the user, the gateway, and the server authenticate each other through the generated random password and device information. If the authentication is not completed or fails within a certain period of time, the search cannot be performed. At the end of successful mutual authentication, both sides of communication establish a secret session key that is further used for future secure communications [31].

## 4. Entity Recognition Method

Because hot entities usually have strong time-varying characteristics and high access requirements, storing entity state information in the edge server will effectively solve the problem of low accuracy caused by the long communication distance. Therefore, this paper proposes a suitable entity recognition method to effectively classify entities on the edge side.

**Figure 2:**
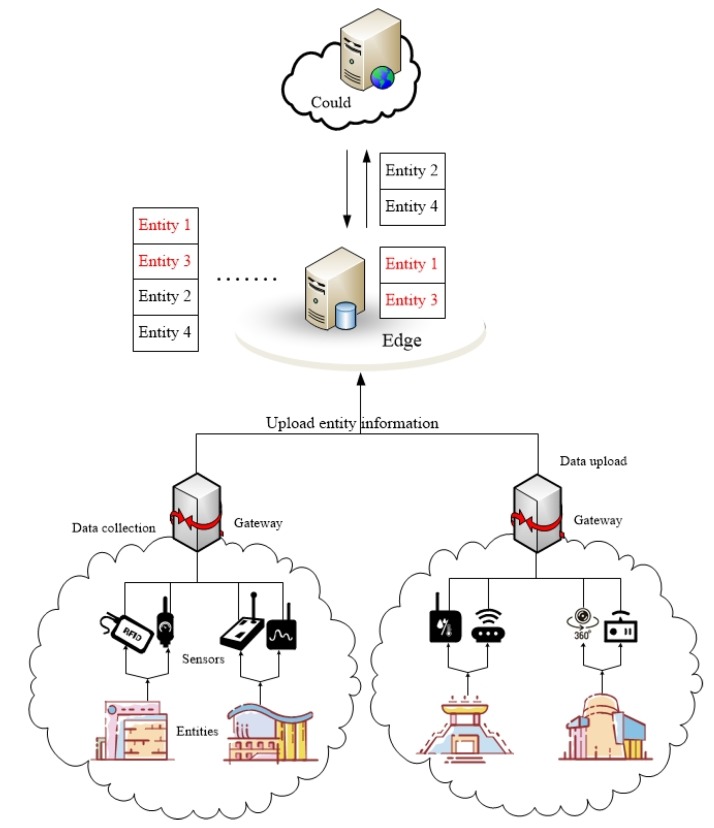
Entity recognition classification storage.

The entity recognition algorithm is executed in the edge server. As shown in Figure 2, for the entities in the coverage area of the edge server, the entity state information uploaded by sensors is collected. For users in the coverage of the edge server, the search records are also recorded. Then identify hot entities and cold entities by adopting the entity classification method based on the multidimensional features of entity. After the entities are classified, the hot entity state information with high access frequency and strong time variability (such as entity 1 and entity 3 in Figure 2) is processed and stored by the edge server. The cold entity state information (such as entity 2 and entity 4 in Figure 2) is uploaded to the cloud.

**Figure 3:**
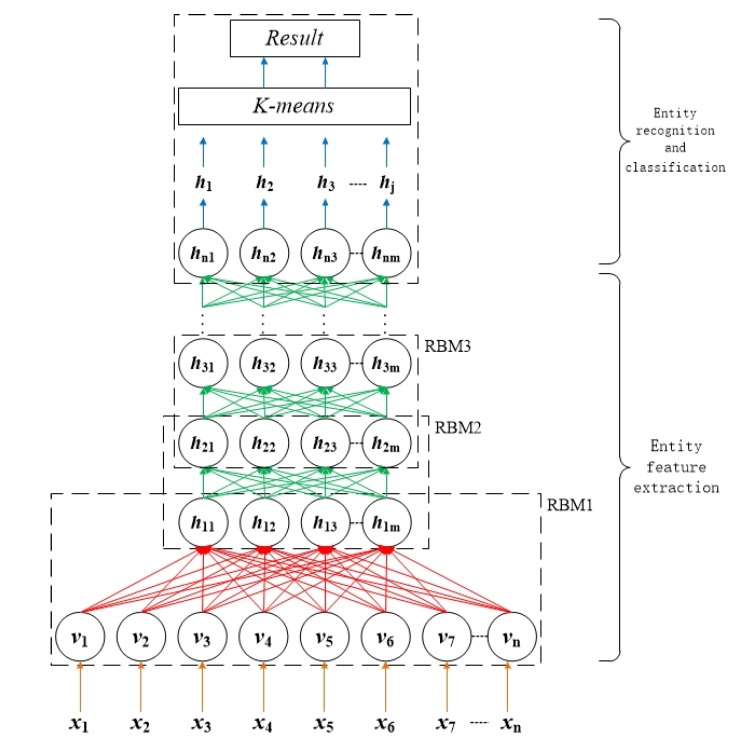
Entity recognition algorithm.

The proposed entity recognition algorithm is based on the Deep Belief Network [32] (DBN) to extract entity state information and user access features, and then uses K-means clustering [33] algorithm to perform unsupervised division of entity categories. The principle of the algorithm is shown in Figure 3. It mainly includes two parts: entity feature extraction and entity recognition classification.

### 4.1. Entity Feature Extraction

Feature extraction [5,34] is the most important part of the entity recognition algorithm. For this algorithm, the entity only needs to be divided into hot entities and cold entities. A single entity usually has multiple features. It is obvious that directly classifying multiple features will obviously affect the accuracy of the classification results. Therefore, through feature extraction, multiple entity features are combined via the relationship between attributes to change the feature space and then achieve the effect of dimensionality reduction, so that can reduce the burden of the classification algorithm and improve the accuracy.

Deep belief network is composed of multi-layer restricted Boltzmann machines [35] (Restricted Boltzmann Machines, RBM). The RBM consists of a visible layer v and a hidden layer h. The visible layer is composed of visible neurons and is used to receive the input entity state features. The hidden layer is composed of hidden neurons and used to extract entity state features. DBN training is performed layer by layer. In each layer, the bottom layer data vectors are used to infer the hidden layer, and this hidden layer is used as the input layer of the next layer.

The feature extraction training steps are as follows:

(1) Fully train the first layer of RBM1.
(2) Fix the weight and offset of RBM1, and then use the state of the hidden neurons as the input vector of RBM2.
(3) After fully training RBM2, stack RBM2 on top of RBM1.
(4) Repeat the above three steps until the DBN converges, to obtain the dimensionality-reduced entity features.

In Figure 3, the input entity characteristics $x_{i}$ include changing frequency, changing amplitude, sampling period, access frequency, number of search times, access time, number of access times and entity ratings. These characteristics can feed back the true status of an entity. For example, change frequency, change amplitude and sampling period can reflect changes in the entity itself. Moreover, number of search times, access time, number of access times and entity ratings can reflect the search and access needs of users for entities. By recombining multiple features, finally, we can obtain features that can reflect the hot/cold conditions of the entity. After the input layer receives the input features, it obtains its neuron state vector $v_{i}$, $v=[v_{1},v_{2},\ldots ,v_{n}]$, hidden neuron state vector $h_{j}$, $h=[h_{1},h_{2},\ldots ,h_{m}]$, *m* is the number of hidden neurons. Given $\left(v,h\right)$, the energy function is

(1) $$E\left(v,h|\theta \right)=-\sum_{i=1}^{n}a_{i}v_{i}-\sum_{j=1}^{m}b_{j}h_{j}-\sum_{i=1}^{n}\sum_{j=1}^{m}v_{i}w_{ij}h_{j}$$

where $\theta =\left\{a,b,w\right\}$ is the set of entity parameters in the RBM, $a_{i}\in a$ is the bias of the neurons in the visible layer, $b_{j}\in b$ is the bias of the neurons in the hidden layer, and $w_{ij}\in w$ is the weight value connecting the visible and hidden layers. The joint probability distribution of RBM under parameter θ is

(2) $$P\left(v,h|\theta \right)=\frac{1}{Z\left(\theta \right)}e^{-E\left(v,h|\theta \right)}$$

where $Z\left(\theta \right)=\sum_{v,h}\exp(-E\left(v,h|\theta \right))$ is the normalization factor. The edge distribution of $\left(v,h\right)$ for *h* is expressed by the function $P\left(v|\theta \right)$ as

(3) $$P\left(v|\theta \right)=\frac{1}{Z\left(\theta \right)}\sum_{h}e^{-E\left(v,h|\theta \right)}$$

### 4.2. Entity Recognition and Classification

After training the entity features through the DBN, the set of entity features $D=\left\{h_{1},h_{2},h_{3},\ldots ,h_{j}\right\}$ can be obtained. To effectively distinguish the category of the entity, thus to determine the storage area of the entity state information, this paper further uses the K-means clustering algorithm to perform cluster analysis on the set of features.

The algorithm process is expressed as follows:

(1) Randomly select $\left\{x_{1},x_{2},x_{3},\ldots ,x_{k}\right\}$ features from the original feature set $D=\left\{h_{1},h_{2},h_{3},\ldots ,h_{j}\right\}$ as the cluster center.
(2) For each remaining solid feature, measure its Euclidean distance to the center of each cluster:
  (4) $$\mathrm{dist}\left(x_{i},x_{k}\right)=\sqrt{{\sum_{d=1}^{D}\left(x_{i,d}-x_{k,d}\right)}^{2}}$$
  It is classified to the nearest cluster center, where D represents the number of original features after processing.
(3) Each iteration of the K-means clustering algorithm needs to update the corresponding cluster center, the mean value of all entity feature in the corresponding cluster is the updated cluster center of the cluster. The update method of the k-th cluster center is
  (5) $$\mathrm{Cente}r_{k}=\frac{\sum_{x_{i}\in C_{k}}x_{i}}{\left|C_{k}\right|}$$
  where $C_{k}$ represents the k-th cluster and $\left|C_{k}\right|$ denotes the number of solid features in the *k*-th cluster.
(4) Iteratively performing step 2 to 3 until the new cluster center is equal to the original cluster center. Finally, the algorithm converges.

After the classification algorithm is completed, a set of hot entities and cold entities are obtained. The edge server stores the hot entity state information locally, and uploads the cold entity state information to the cloud server.

## 5. Interest Group-Based Collaborative Filtering Method

To meet the potential needs of users, ensure the quality of the recommendation system, and recommend entity state information that users are interested in. This paper further proposes an interest group-based collaborative filtering (IGCF) recommendation algorithm. The algorithm first determines the number of interest groups [36,37,38,39] through the Canopy clustering algorithm, and then divides multiple interest groups by adopting the K-means clustering algorithm. Measure the similarity of users in the interest group according to the Pearson correlation coefficient. Find the user’s related nearest neighbors and obtain the candidate set recommended by the entity to generate the recommendation results.

### 5.1. Interest Group Division

K-means clustering algorithm, as a commonly used clustering algorithm, has obvious advantages, the algorithm has fast convergence speed, and the clustering effect is excellent. However, it also owns some disadvantages. The clustering results depend on the selection of cluster numbers and are sensitive to noise and outliers. Compared with the K-means algorithm, the Canopy [40] algorithm does not need to determine the number of clusters in advance, the algorithm is fast, and the smaller NumPoint Cluster can be removed during the clustering process, which improves the anti-interference ability of subsequent algorithms, but the accuracy is lower. Therefore, we can use the Canopy algorithm to perform coarse clustering to obtain the K value required by the K-means algorithm, and then use the K-means algorithm to obtain the final clustering result.

The algorithm process is expressed as follows:

(1) Define T1 and T2 as thresholds of Canopy radius through cross-validation, where T1 > T2.
(2) Randomly select a data x from the data set P as the center of the first Canopy, and delete x from P.
(3) Calculate the distance d between the remaining data in P and x.
(4) If it is d < T1, mark the data as a weak association and divide the data object into Canopy. At this time, the data can still be used as the center of another Canopy. If d < T2, the data object is marked as a strong association, and it is deleted from P.
(5) Repeat steps (2)–(4) until P is empty and the algorithm converges.

**Figure 4:**
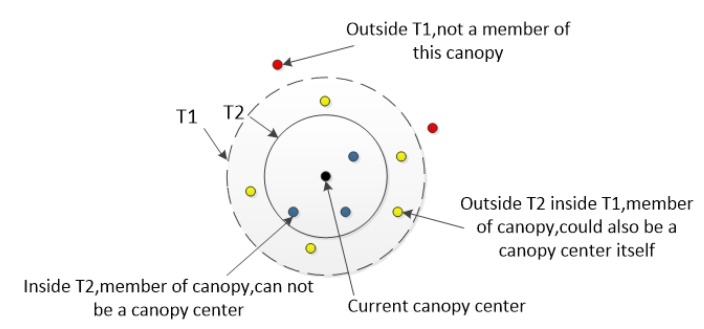
Canopy algorithm.

Canopy refers to a subset of similar objects stored together by calculating the similarity of sample data in data set. As shown in Figure 4, a random data is used as the center of the circle, T1 is the radius of the large circle, and T2 is the radius of the small circle. The data items in the small circle must belong to this Canopy and cannot be used as the center of another Canopy. The data points between the big circle and the small circle are considered to belong to this Canopy, but can still be used as the center of a new Canopy to divide the data. The data points outside the big circle do not belong to this Canopy. The number of Canopy obtained is used as the K value of the K-means algorithm, and the result of the interest group division is finally obtained through the K-means algorithm.

### 5.2. User Similarity Calculation

In collaborative filtering system, only by measuring the similarity between users or between objects, can we find the nearest neighbor and complete the recommendation. Commonly used similarity calculation methods include cosine similarity, Euclidean distance, and Pearson correlation coefficient. Cosine similarity is the most universally used similarity calculation method and is widely used to calculate the similarity of document data. It uses the cosine value of the angle between two n-dimensional vectors to compare the differences between two objects. However, the result is inaccurate because it is not sensitive to numerical values. Euclidean distance refers to the true distance between two points in the m-dimensional space, but relies on the common rating item. If there is no common rating item, the two users or items are considered to be completely different. Pearson correlation coefficient measures the closeness of the relationship between variables, and it is suitable for processing scoring data, and performs well in user-oriented collaborative filtering. Therefore, we adopt Pearson correlation coefficient to compute the similarities of users in interest groups.

The Pearson correlation coefficient reflects the degree of correlation between two users, and its value is between [−1, 1]. When the correlation between two users increases, the correlation coefficient tends to 1 or −1; a correlation coefficient greater than 0 indicates a positive correlation between the two users; a correlation coefficient less than 0 indicates a negative correlation between the two users; If the correlation coefficient is equal to 0, it indicates that there is no correlation between users.

The Pearson correlation coefficient is defined as the quotient of the covariance and standard deviation between two users. which is

(6) $$\begin{array}{cc} \rho_{XY} & \;=\;\frac{\mathrm{Cov}(X,Y)}{\sigma_{X}\sigma_{Y}}\\ & \;=\;\frac{E((X-\mu X)(Y-\mu Y))}{\sigma_{X}\sigma_{Y}} \end{array}$$

where $E\left(X\right)=\sum_{i=1}^{n}X_{i}P\left(X_{i}\right)$, μX=E(X),

(7) $$\begin{array}{cc} \sigma_{X} & =\sqrt{D(X)}\\ & =\sqrt{E({\left(X-E(X)\right)}^{2})}\\ & =\sqrt{E\left(X^{2}\right)-E^{2}\left(X\right)} \end{array}$$

Finally, the Pearson correlation coefficient formula between users is

(8) $$\rho_{XY}=\frac{\sum_{i=1}^{n}\left(X_{i}-\bar{X}\right)\left(Y_{i}-\bar{Y}\right)}{\sqrt{{\sum_{i=1}^{n}\left(X_{i}-\bar{X}\right)}^{2}}\sqrt{{\sum_{i=1}^{n}\left(Y_{i}-\bar{Y}\right)}^{2}}}$$

where i∈R(U,I), *R* is a rating set of user-entity, $X_{i}$ is the rating given by user *X* to entity *i*, $\bar{X}$ is the average rating given by user *X* to all entities. Similarly, $Y_{i}$ is the rating given by user *Y* to entity *i*, $\bar{Y}$ is the average rating given by user *Y* to all entities. The correlation distance between *X* and *Y* is calculated as $D_{XY}=1-\rho_{\mathrm{XY}}$. Finally, the nearest neighbor set *N* of user *X* is obtained according to the Pearson correlation coefficient.

Then, predict the rating of user *X* to entity *e* which has not been searched by user *X* based on the other users in the nearest neighbor set *N*

(9) $$\mathrm{pred}\left(X,e\right)=\bar{X}+\frac{\sum_{Y\in N}\rho_{XY}\left(Y_{e}-\bar{Y}\right)}{\sum_{Y\in N}\rho_{XY}}$$

At last, a list of recommended entities is obtained according to the prediction results, and the recommendation is completed.

## 6. Simulation Verification and Result Analysis

To verify the reactive recommendation method, we use Intellab [41] data set. This data set contains data collected and uploaded by 54 temperature and humidity sensors deployed at different locations. The number of samples is 10,000 per sensor. The simulation environment is 64-bit Windows 10 operating system, the processor is Intel (R) Core (TM) i5-3337U, 8GB memory, CPU 1.80GHz, and the development tools are MATLAB R2017b and Python. The DBN parameter settings are shown in Table 1. In K-means, the K value is set to 2. The performance verification results are average values after 1000 searches.

To verify the active recommendation method, the data set used is Yelp’s [42] real check-in data set, which contains score data and check-in times for many check-in places such as restaurants, hotels, cafes, and bars. Filter out the check-in city in the dataset for Phoenix, check-in location information with more than 20 reviews, and user rating information for those places that have been checked in and visited. There is a total of 31,302 pieces of data. To compare the performance of the algorithm, 80% of the data set is randomly divided as training data and 20% is divided as test data.

### 6.1. Entity Recognition Algorithm Based on Deep Belief Network

**Figure 5:**
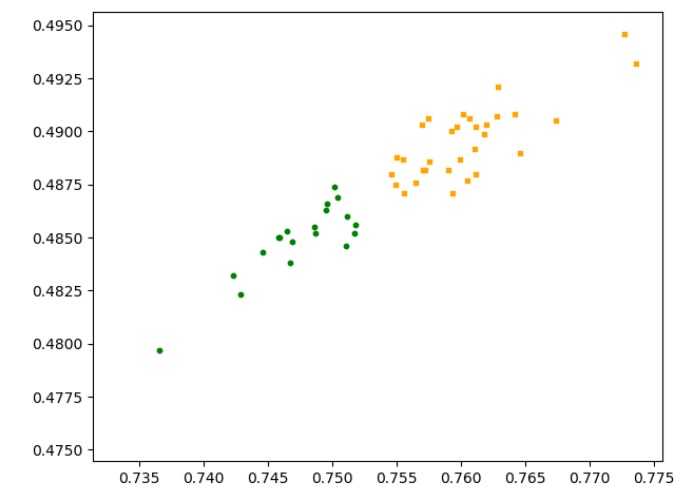
Classification result of the entity recognition algorithm based on deep belief networks.

**Figure 6:**
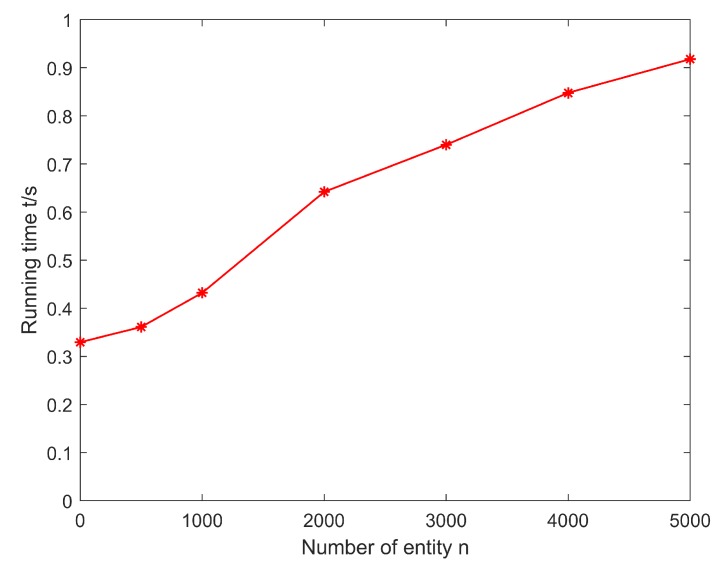
Running time of the algorithm in different numbers of entities.

Figure 5 is the classification result of the entity recognition algorithm based on deep belief networks. It can be seen that after the algorithm extracts the entity features, the entities are divided into two different types of entities, namely hot entities and cold entities. Figure 6 shows the running time of the entity recognition algorithm based on deep belief networks in different numbers of entities. It can be seen that as the number of entities increases, the runtime of the algorithm also increases. This is because the increase in the number of entities leads to an increase in computing cost, which results in longer calculation times.

### 6.2. Precision and Recall

**Figure 7:**
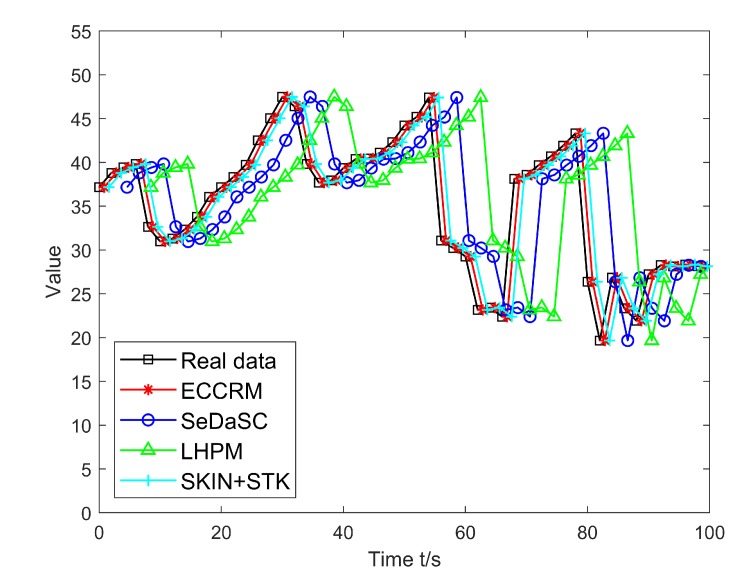
Comparison of search results for hot entities from cloud and edge servers.

After the entity recognition algorithm based on the Deep Belief Network classifies the entities, the simulation is performed on the strongly time-varying entities. Figure 7 is a comparison of the results obtained by performing ECCRM, SKIN+STK and SeDaSC after storing the hot entity status information on the edge server and the cloud server. The average error of the data searched by the ECCRM and SKIN+STK are 1.2005 and 2.0086, while the average errors of the data searched by the SeDaSC and LHPM are 5.2708 and 8.2119. It can be seen that for strongly time-varying entities, results obtained after being stored on the edge server are far more accurate than those stored in the cloud due to communication delays.

**Figure 8:**
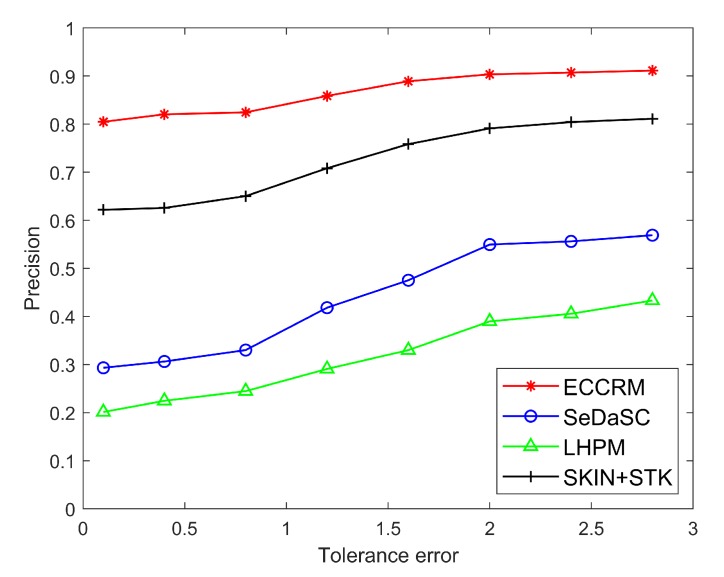
Comparison of precision of hot entities under different tolerance errors.

**Figure 9:**
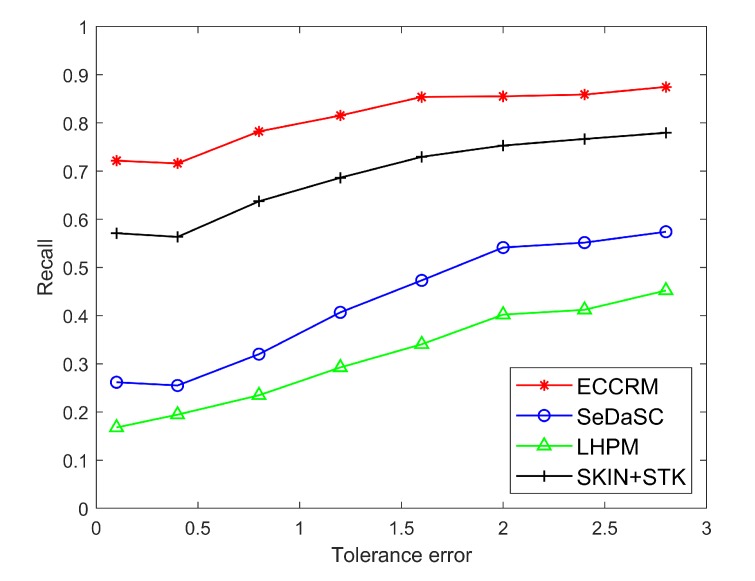
Comparison of recall of hot entities under different tolerance errors.

Figure 8 and Figure 9 are comparisons of the precision and recall of hot entities stored on edge servers and the cloud under different user tolerance errors.

Precision is defined as:

(10) $$\mathrm{Precision}\;=\;\frac{\left|s_{2}\right|}{\left|s_{1}\right|}$$

Recall is defined as:

(11) $$\mathrm{recall}\;=\;\frac{\left|s_{2}\right|}{\left|s_{3}\right|}$$

where $S_{1}$ is the set of sensor search results after giving the range of search sensor values, $S_{2}$ is the set of sensors whose true values in the given range are in the search result set $S_{1}$, set $S_{3}$ is all the sensors whose true values are within the given range set.

It can be seen that due to the strong time variability of hot entities and the short delay in searching data from the edge server, the results are closer to the real data. Therefore, the precision and recall of the hot entity status information after searching from the edge server are much higher than cloud. Moreover, As the user’s tolerance for results increases, the precision and recall of searches often increases slightly. This is because as the tolerance error increases, the range of values allowed in the results also increases, resulting in an increase in the number of sensors in the set, which leads to an increase in the precision and recall.

**Figure 10:**
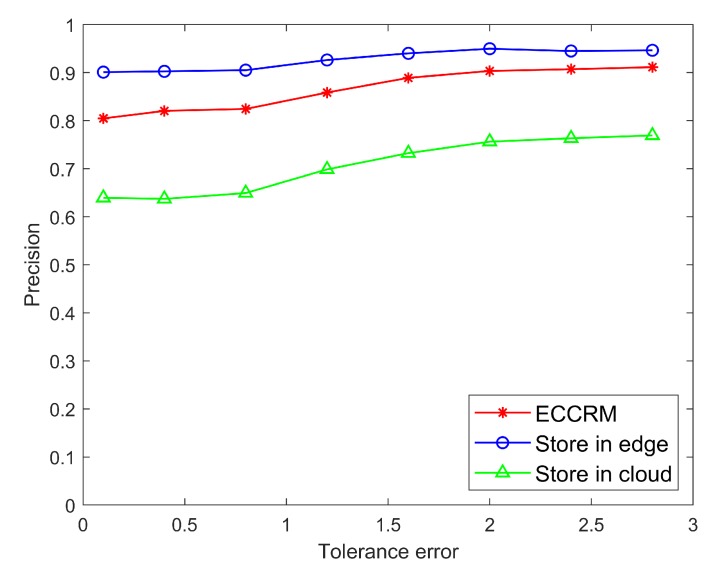
Comparison of precision of different store scheme under different tolerance errors.

**Figure 11:**
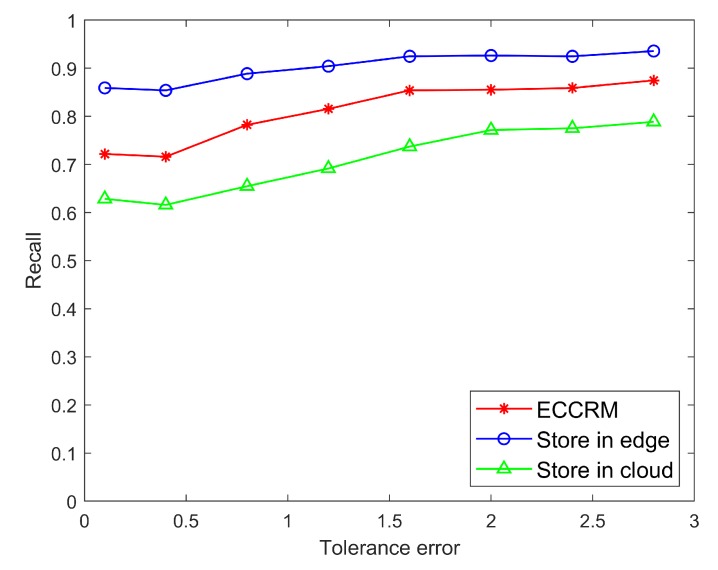
Comparison of recall of different store scheme under different tolerance errors.

Figure 10 and Figure 11 are comparisons of the precision and recall of different entities store scheme under different user tolerance errors. It can be seen that when all data under not classified and stored in the edge, the precision and recall are the highest. Because the short delay in searching data from the edge server, the search results are closer to the real data. Because the data stored in the cloud contains cold entities with weak time variability, it ensures the precision and recall of cloud storage solutions has improved. However, centralizing the storage of data consumes a lot of computing and storage resources. Due to the weak time variability of cold entities, there is no significant difference in search results when entities stored on the edge or in the cloud. However, searching for the hot entities requires very timely feedback. Therefore, it is unwise to store all data on the edge or in the cloud, which will cause unnecessary waste of computing and storage resources. While our algorithm was designed with consideration of how to save bandwidth and storage resources. In our scheme, storing hot entity information in the edge can save the communication bandwidth of uploading data to the cloud and the storage resources of the cloud. Moreover, recommending entity information through our recommendation algorithm can save the search spending of the user, so as to save the communication bandwidth between users and servers. In summary, our scheme is more applicable than other schemes.

### 6.3. Algorithm Performance Verification

**Figure 12:**
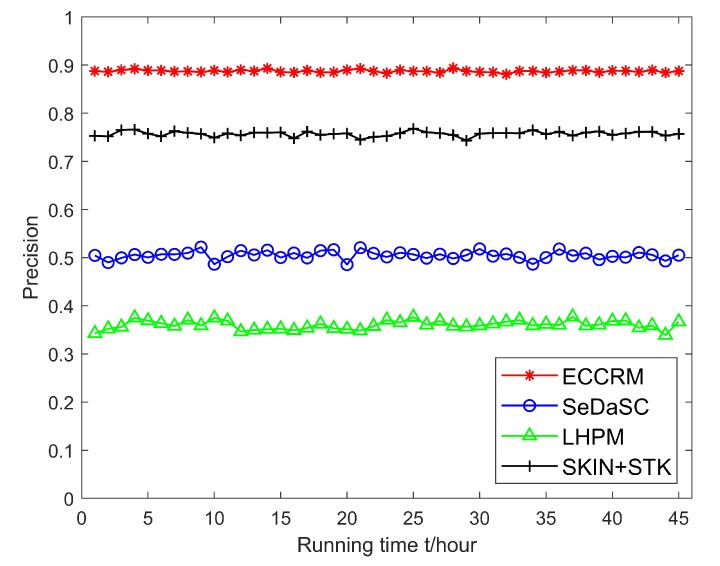
Comparison of precision.

**Figure 13:**
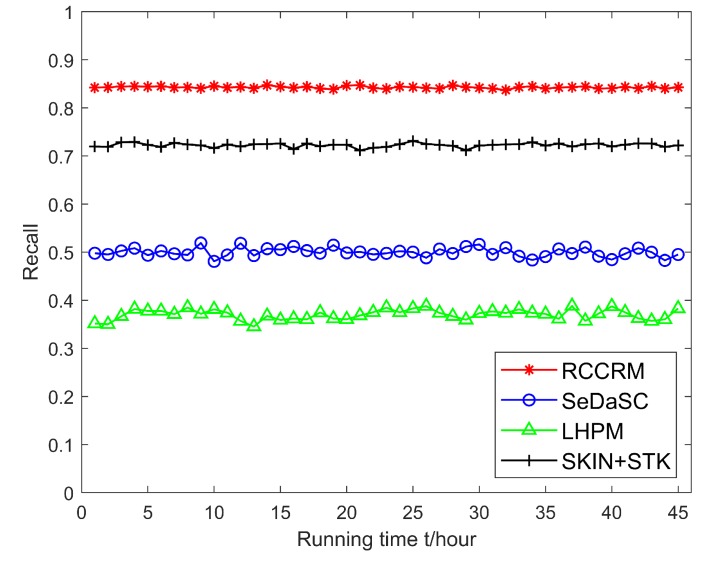
Comparison of recall.

Figuress Figure 12 and Figure 13 show the precision and recall of the results as the algorithm’s running time increases. It can be seen that the stability of the precision and recall of the ECCRM scheme is higher than SKIN+STK, SeDaSC and LHPM schemes. This is due to the strong time variability of hot entities. In ECCRM scheme, because the edge server is close to the user, the delay in results from the edge server is relatively short. The matching rate between search results and the current state of the entity is higher, so the precision and recall are less volatile; In the SeDaSC scheme, the delay of long-distance communication is large. Before user obtains the results, the probability of the entity state changing is relatively high, so the results do not match the current state of the entity, which makes the precision and recall fluctuate significantly. In the LHPM scheme, after the gateway receives and parses the user’s request, it needs to access the sensors matched by the search request and obtain data. Because the communication distance is too long, the entity state has changed when the results are returned, so the accuracy rate and recall rate fluctuate greatly. It is worth noting that even SKIN+STK scheme stores data at the edge, but it must decide whether relevant search results can be directly returned from n-hop neighbor regions when searching. If not, it needs to access the corresponding sensor to get search results, thus the accuracy rate and recall rate fluctuate greatly.

**Figure 14:**
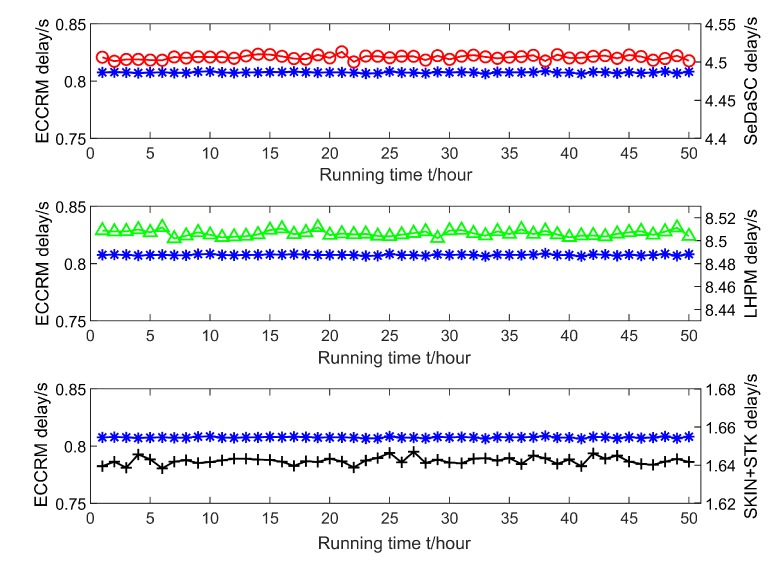
Comparison of algorithm stability.

Figure 14 shows the fluctuations in the latency of searching data from the edge server and the cloud server as the running time of the algorithm increases. It can be seen that with the increase of the algorithm running time, the fluctuation of the time-consuming delay of the ECCRM scheme are smaller than the SKIN+STK scheme, the SeDaSC scheme and the LHPM scheme. This is because the communication distance between the user and the edge server is short, and the communication is more stable, so the fluctuation of delay is smaller. The communication distance between the cloud server and the user is too long. The number of terminals communicating with the cloud server at the same time is huge, and the probability of congestion or queuing is higher, so the fluctuation of delay is small. The SKIN+STK scheme is very special. It never uses cloud server to store data, but obtains data from nearby n-hop edge servers. The delay increases with the number of hops, thus the delay fluctuates significantly.

### 6.4. Interest Group Division

**Figure 15:**
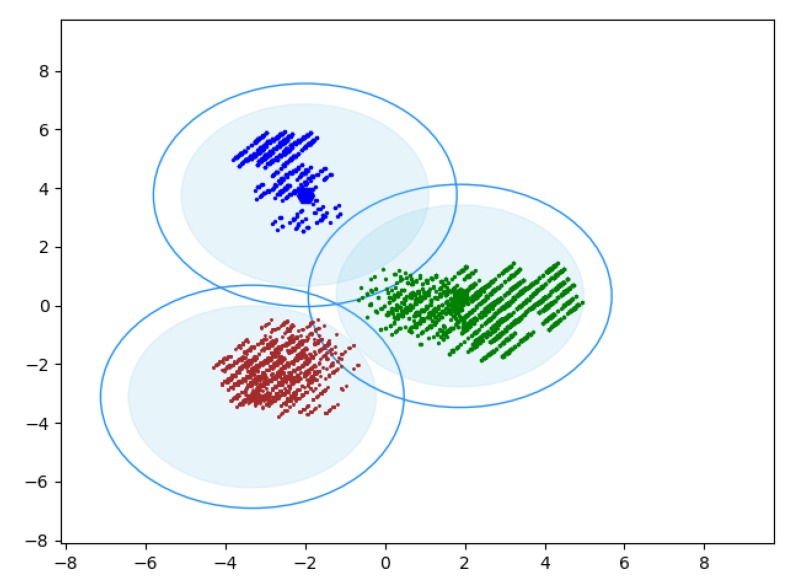
Result of Canopy algorithm.

**Figure 16:**
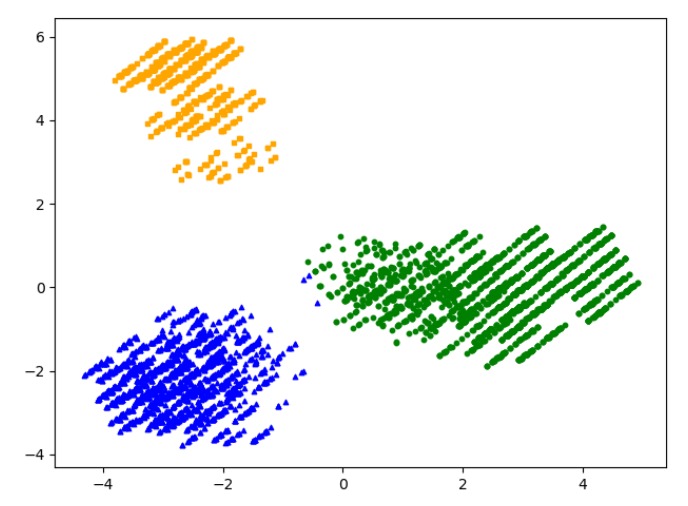
Result of Interest group division.

**Figure 17:**
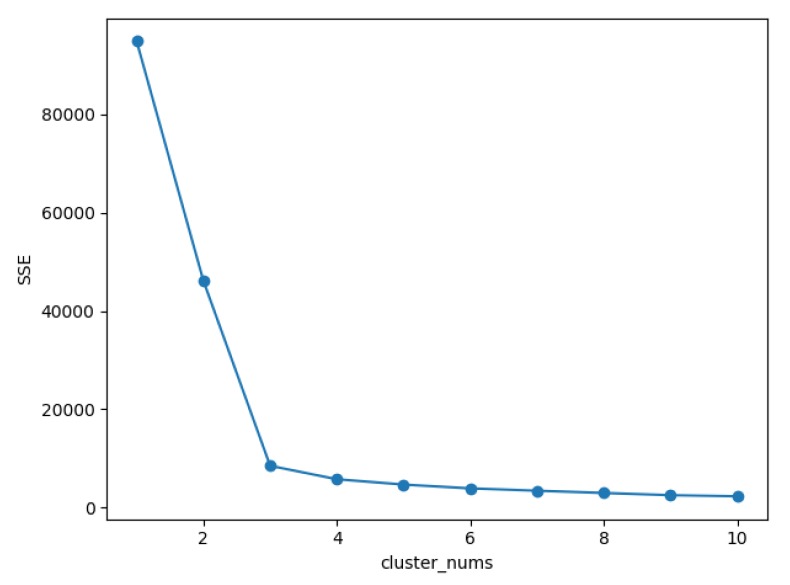
SSE at different K values.

Figure 15 shows the simulation results of clustering by the Canopy algorithm. It can be seen that the algorithm obtained 3 Canopies, so the K value of the K-means clustering algorithm was determined as 3. Figure 16 shows the results of the K-means clustering algorithm. It can be clearly seen that the user set has been divided into three interest groups. To ensure that the selection of K value is correct, calculated the within cluster Sum of Squared Errors (SSE) at different K values, determine the choice of K’s value through the Elbow Method [43]. It can be seen from Figure 17 that when K = 3, the distortions are the largest, so that the choice of K’s value is correct.

To evaluate the performance of the recommendation system, the performance of the recommendation system is measured by Mean Absolute Error (MAE), Mean Square Error (MSE), and Root Mean Square Error (RMSE).

MAE reflects the error of the prediction result. The smaller the MAE, the better the effectiveness of the recommendation system. The definition of MAE is

(12) $$\mathrm{MAE}=\frac{1}{N}\sum_{i=1}^{N}\left|\mathrm{rea}l_{i}-\mathrm{pre}d_{i}\right|$$

MSE reflects the degree of difference between the predicted value and the true value. The smaller the MSE value, the higher the accuracy of the recommendation system. It is defined as

(13) $$\mathrm{MSE}=\frac{1}{N}\sum_{i=1}^{N}{\left(\mathrm{rea}l_{i}-\mathrm{pre}d_{i}\right)}^{2}$$

RMSE is based on MSE. To avoid the impact of the dimension on the results, it can better describe the quality of the recommendation system. The smaller the value of RMSE, the more accurate the recommendation system is. It is defined as

(14) $$\mathrm{RMSE}=\sqrt{\frac{1}{N}\sum_{i=1}^{N}{\left(\mathrm{rea}l_{i}-\mathrm{pre}d_{i}\right)}^{2}}$$

where *N* is the number of test samples, $\mathrm{rea}l_{i}$ is the user’s true rating for entity *i*, and $\mathrm{pre}d_{i}$ is the user’s predicted rating for entity *i*.

To measure the advantages and disadvantages of the proposed algorithm, the algorithm proposed in this paper is compared with the following algorithm:

(1) User-based collaborative filtering (UCF) [44],which measures the similarity of users in the user-entity matrix through the cosine similarity, then get the recommended results.
(2) Matrix factorization (MF) [45] -based recommendation algorithm obtains a new matrix by reducing the dimension of the user-entity matrix, and then recommends the results of the user’s ranking of the entities in the new matrix.
(3) Regression-based collaborative filtering(RCF). This algorithm predicts the user’s rating on an entity based on the linear regression, optimizes the offset value of the algorithm by alternating least squares, and makes recommendations based on the prediction results of the score.

In the rating prediction, the number of the neighbors will directly affect the quality of the recommendation system. Too many neighbors will bring extra noise to the system. Too few neighbors will negatively affect the quality of the recommendation system. Therefore, we mainly simulate the performance of each recommendation algorithm under different numbers of neighbors, find the optimal number of neighbors and compare the results.

**Figure 18:**
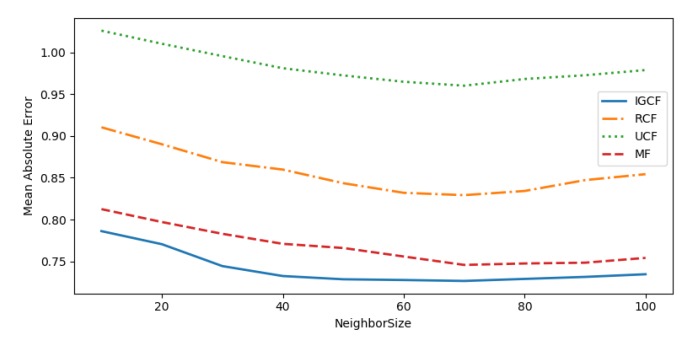
MAE.

**Figure 19:**
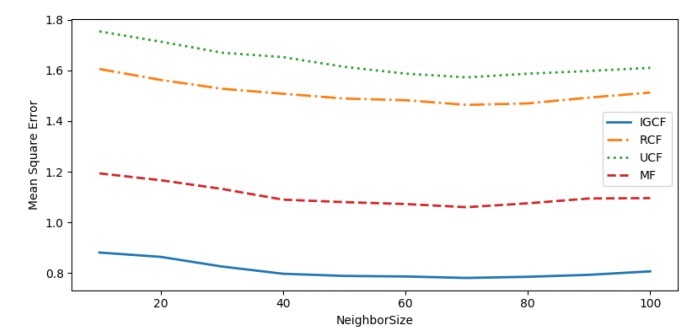
MSE.

**Figure 20:**
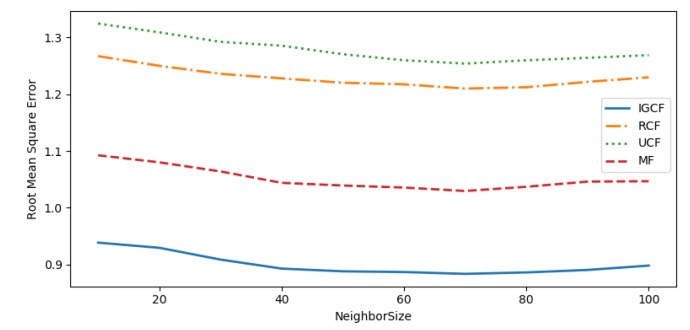
RMSE.

As can be seen from Figure 18, Figure 19 and Figure 20, the recommended accuracy of the IGCF algorithm proposed in this paper is higher than the other algorithms, and each algorithm performs best when NeighborSize = 70. The UCF algorithm obtains the nearest neighbors of similar users by calculating the cosine similarity, while the cosine similarity is not sensitive to absolute values, and the error calculated in the scoring matrix is large, so the result is the worst. The RCF algorithm predicts the user’s rating of an entity based on the linear regression. When the user’s rating data is sparse, the error of the prediction result will increase, so the effect is not good. The MF algorithm takes into account the sparseness of the scoring matrix, and obtains a more efficient matrix through dimension reduction before measuring the relevance of the user, so it is better than the UCF algorithm and the RCF algorithm. However, because it is the correlation of all users in the calculation matrix, errors are easy to occur. The user group is not considered to be prioritized, and the similarity between users is calculated in the divided user group to reduce errors. Therefore, the MF algorithm performs worse than the algorithm proposed in this paper. Table 2 shows the comparison of algorithm results when NeighborSize = 70.

## 7. Conclusions

In this paper, in view of the high real-time requirements of IoT search and the strong time-varying characteristics of entities in the physical world, combining the respective advantages of cloud computing and edge computing, an edge-cloud collaboration entity recommend method for IoT search is designed to improve the search accuracy and search efficiency of physical entities. Considering the limited communication capabilities of sensors embedded in physical entities, this paper proposes an entity recognition algorithm based on Deep Belief Network set in edge server, which can distinguish hot and cold entities. By storing the hot entity status information in the edge server and the cold entity status information in the cloud, algorithm saves the storage space and computing overhead of the edge server, while ensuring the timeliness and accuracy of the recommendation results. Considering the user’s interest preferences and potential search needs, this paper proposes an interest group-based collaborative filtering recommendation algorithm set in the cloud server. By dividing user interest groups, recommending the entity status information that the user is interested in into users in interest group, thereby improving the intelligence level of the recommendation system.

## Figures and Tables

**Table 1:** DBN parameter settings.

Parameters	Numerical Value
RBM layers	3
Learning rate	1
Random samples	10
Number of neurons in the first layer	5
Number of neurons in the second layer	2
Number of neurons in the third layer	2

**Table 2:** Comparison of algorithm results when NeighborSize = 70.

Algorithm	MAE	MSE	RMSE
UCF	0.9602	1.5720	1.2538
RCF	0.8292	1.4636	1.2098
MF	0.7457	1.0601	1.0296
IGCF	0.7265	0.7811	0.8838
